# Editorial: Recent Developments in Micron-Scale Optical Imaging of Intact, Living Heart and Vasculature

**DOI:** 10.3389/fphys.2016.00490

**Published:** 2016-10-26

**Authors:** Gil Bub, Michael Rubart

**Affiliations:** ^1^Physiology Department, McGill UniversityMontreal, QC, Canada; ^2^Riley Heart Research Center, Department of Pediatrics, Wells Center for Pediatric Research, Indiana University School of MedicineIndianapolis, IN, USA

**Keywords:** intravital, microscopy, fluorescence, cardiac, vasculature, photonics

While the behavior of all cells depends on their environment, this is especially true in the heart and vasculature where tissues generate and respond to large gradients in mechanical forces and rapid changes in membrane voltage. At the same time, these features pose unique challenges for scientists imaging the dynamics of cells at microscopic scales *in situ*. The collection of papers in this special issue discusses several strategies for *in-situ* micron-scale optical imaging of the heart and vasculature, ranging from the use of novel probes to new microscopy techniques and analysis methods.

A major focus of many authors was the development of novel microscopy tools or the use of existing commercial systems in new ways. Vinegoni et al. review recent work done by their lab and others in *in-vivo* (intravital) cardiac microscopy, where one major challenge is immobilization of the heart without using pharmacological approaches. Hou et al. describe modifications in the light path of a spinning disk confocal microscope that improves throughput at the expense of uniform illumination, allowing functional imaging of endogenous probes. Chen et al. combine conventional fluorescence based optical techniques, video motion tracking and atomic force microscopy to measure the dynamics of the developing murine heart, and show that atomic force microscopy detects contraction in embryonic hearts before they become detectable using other approaches. Crocini et al. review work by their group and others' on the use of acousto-optical deflectors (AODs) instead of galvanometers for steering lasers during scanning laser microscopy: Here, they show that AOD-based systems can be used to capture voltage transients from several t-tubules within a single cardiomyocyte in under a millisecond. Two groups discuss distortions in optical measurements of cardiac tissue, arising either from the architecture of the imaging system itself or the interaction of small anatomical features of the tissue with light: Corbett et al. describe potential image distortions in remote focusing microscopy, a relatively new imaging modality that allows fast scanning in the axial direction, and Bishop and Plank use photon scattering simulations to show that humps in the action potential waveform can be caused by vasculature perturbing photon light paths. This issue also has two reviews on measuring functional and structural properties of living hearts using conventional tools. Chen et al. give a detailed description of a setup for imaging cardiac t-tubule networks in Langendorff-perfused whole heart using single photon confocal microscopy in the context of t-tubule remodeling in heart failure and infarcted hearts. Lu and Rubart review techniques for functional imaging using one- and two-photon confocal microscopy in the Langendorff-perfused heart, with a focus on measuring the voltage and calcium dynamics from clinically relevant populations of exogenously added cardiac cells (e.g., stem cells). Finally, Dura et al. summarize the state of the art in cardiac mapping experiments where the goal is to measure fluorescence signals over the entire surface of the mouse heart. Here attention is given to algorithmic approaches and optical techniques needed for panoramic imaging and reconstruction of accurate maps, with a focus on challenges associated with scaling techniques that are used in larger preparations, to the smaller murine heart.

Several authors in this issue discuss their research on the use and development of exogenous and endogenous probes designed for measuring events in the heart and vasculature. Shui et al. present a review summarizing the work done by the Cornell Heart Lung Blood Resource for Optogenetic Mouse Signaling (CHROMus), which aims to develop transgenic mouse lines for combinatorial crosses for the simultaneous expression of fluorescence sensors and optogenetic based effectors. CHROMus is an ongoing project with nine mouse lines available and many more in development. Hou et al. paper introduces a newly developed dual calcium/voltage reporter (CaViar), which combines the far red voltage indicator Arch (D95N) and the blue/green calcium indicator GCaMPP5G. The authors use this probe in a modified spinning disk confocal system to simultaneously map voltage and calcium transients from a zebra fish line and find distinct changes in action potential morphologies during development. Huang et al. describe new methods for localized dye loading when using fiber-optic based confocal microscopes (FCM), which are often used in clinical settings where systemic loading isn't desirable. The manuscript by Thunemann et al. presents a methods paper detailing the use of intravital ratiometry and two photon microscopy in transgenic mice models expressing a FRET-based cGMP sensor for measuring vasodilation. In the same vein, Fairfax et al. published original research on measuring calcium signaling on the vasculature of conscious mice expressing a smooth muscle-specific FRET-based calcium indicator, the first demonstration of calcium measurement in smooth muscle of arterioles in conscious animals.

Several papers leverage one of the defining features of the healthy heart—its periodicity—to improve temporal resolution. Vinegoni et al. review the use of retrospective gating (where data is re-ordered after acquisition) and prospective triggering (where the sensor is triggered off a periodic stimulus used to drive the heart) in their intravital cardiac studies for measuring cardiomyocyte structure. Taylor, in addition to reviewing gating methodologies, discusses his work on optical gating where fast video and computational methods are used predict the position of the heart in close to real time, which is necessary for gating in smaller intact animals such as the zebrafish. These approaches are also leveraged for functional studies: Hou et al. use data from several heart beats to increase the effective temporal resolution of captured calcium transients, and Hammer et al. used a temporal registration-based analysis method to increase the temporal resolution of a 55 fps spinning disk confocal system to over 300 fps to measure variations in calcium transients at the single cell level.

It is hoped that this collection of papers provides a snapshot of the current state of the art in this rapidly evolving field, and may function as a jumping-off point for researchers interested in measuring the dynamics and structure of cardiac cells *in-situ*.

## Author contributions

All authors listed, have made substantial, direct and intellectual contribution to the work, and approved it for publication.

### Conflict of interest statement

The authors declare that the research was conducted in the absence of any commercial or financial relationships that could be construed as a potential conflict of interest.

